# Pazopanib in Metastatic Renal Cancer: A “Real-World” Experience at National Cancer Institute “Fondazione G. Pascale”

**DOI:** 10.3389/fphar.2016.00287

**Published:** 2016-08-31

**Authors:** Sabrina C. Cecere, Sabrina Rossetti, Carla Cavaliere, Chiara Della Pepa, Marilena Di Napoli, Anna Crispo, Gelsomina Iovane, Raffaele Piscitelli, Domenico Sorrentino, Gennaro Ciliberto, Piera Maiolino, Paolo Muto, Sisto Perdonà, Massimiliano Berretta, Sandro Pignata, Gaetano Facchini, Carmine D'Aniello

**Affiliations:** ^1^Division of Medical Oncology, Department of Uro-Gynaecological Oncology, Istituto Nazionale Tumori IRCCS “Fondazione G. Pascale,”Naples, Italy; ^2^Department of Onco-Ematology Medical Oncology, S.G. Moscati Hospital of TarantoTaranto, Italy; ^3^Unit of Epidemiology, Struttura Complessa di Statistica Medica, Biometria e Bioinformatica, Istituto Nazionale Tumori IRCCS “Fondazione G. Pascale,”Naples, Italy; ^4^Pharmacy Unit, Istituto Nazionale Tumori IRCCS “Fondazione G. Pascale,”Naples, Italy; ^5^Division of Urology, Department of Uro-Gynaecological Oncology, Istituto Nazionale Tumori IRCCS “Fondazione G. Pascale,”Naples, Italy; ^6^Scientific Direction, Istituto Nazionale Tumori IRCCS “Fondazione G. Pascale,”Naples, Italy; ^7^Division of Radiation Oncology, Istituto Nazionale Tumori IRCCS “Fondazione G. Pascale,”Naples, Italy; ^8^Division of Medical Oncology–Centro di Riferimento OncologicoAviano, Italy; ^9^Oncology Unit, A.O.R.N. dei COLLI “Ospedali Monaldi-Cotugno-CTO,”Naples, Italy

**Keywords:** mRCC, first-line, pazopanib, real-life, PFS

## Abstract

Pazopanib is an oral angiogenesis inhibitor, currently approved for treatment of metastatic renal cell carcinoma (mRCC) and soft tissue sarcoma. The present study analyzed the outcomes of pazopanib in first-line treatment of mRCC, in a single Italian cancer center. In the light of the retrospective, observational nature and the unselected population, our experience can be defined a “real-world” study. The medical records of 38 mRCC patients treated with front-line pazopanib were retrospectively reviewed and analyzed. The progression free survival (PFS) and the overall survival (OS) were the primary endpoints, while secondary objectives included objective response rate (ORR), disease control rate (DCR), and treatment tolerability. Pazopanib achieved a median PFS (mPFS) of 12.7 months (95% CI, 6.9–18.5 months). The median OS (mOS) was 26.2 months (95% CI, 12.6–39.9 months); the observed ORR and DCR were 30.3 and 72.7%, respectively, with a median duration of response of 11 weeks. mPFS appeared not to be influenced by number of co-morbidities (< 3 vs. ≥3), gender, Fuhrman grade and age. Conversely, the ORR and the DCR positively affect the mPFS (HR = 0.05 [95% CI, 0.05–0.55], *p* = 0.01; HR = 0.10 [95% CI, 0.02–0.43], *p* = 0.002, respectively). A worse outcome was associated with a lower mPFS in patients with liver metastases (*p* = 0.2) and with a high tumor burden (number of metastatic sites < 6 vs. ≥6) (*p* = 0.08). Worst OS was observed in patients aged ≥70 years old (HR = 6.91 [95% CI, 1.49–31.91], *p* = 0.01). The treatment was well-tolerated: no grade 4 adverse events, nor discontinuation due to toxicities was reported. Grade 3 hypertension affected positively the OS reaching the statistical significance (HR = 0.22 [95% CI, 0.05–0.8], *p* = 0.03). Thyroid dysfunction (hypo and hyperthyroidism) seems to correlate with better outcome in terms of a longer mPFS (HR = 0.12 [95% CI, 0.02–0.78], *p* = 0.02). Our results are consistent with those reported in prospective phase III trials and the published retrospective “real world” experiences. This analysis confirms the safety and efficacy of pazopanib in first-line setting, both in frail patients with multiple co-morbidities and Karnofsky PS < 80% and in younger, healthier patients with a number of metastatic sites < 6.

## Introduction

Renal cell carcinoma (RCC), is the most common subtype of kidney cancer (Choueiri, 2011). Approximately 90% of these tumors are classified as RCC and up to 80% as “clear cell” RCC (Janzen et al., 2003; Choueiri, 2011). Most patients (>75%) are diagnosed when the disease is already advanced or metastatic, mRCC, hence no more curable (Facchini et al., 2009). Recurrent and/or mRCC is associated with a poor 5-years survival, roughly 10%; however, in the last decade, a series of novel agents have been introduced in clinical practice and the outcomes are slowly improving. Despite an increasing knowledge about the genetic and signaling abnormalities involved in the RCC carcinogenesis, such as *VHL, PBRM1, SETD2*, and *BAP1* genes, no biomarkers are currently available, thus there are no molecular factors, which may guide the clinicians in choosing the therapeutic strategy (Brugarolas, 2014).

The advent of the target therapies (TTs) has revolutionized the mRCC treatment with an impressive effect on the overall survival (OS), which increased from an average of 9 months in 1995, when the only option in first-line was interferon alfa (IFN-α), to a median of 28–29 months in 2013, the TTs era (Motzer and Russo, 2000; Chowdhury et al., 2008; Albiges et al., 2015; de Velasco et al., 2015). The TTs include the tyrosine kinase inhibitors (TKIs), targeting the vascular endothelial growth factor receptors (VEGFRs), the mammalian target of rapamycin inhibitors (mTORis), and bevacizumab, a monoclonal antibody targeting the VEGF ligand.

Sunitinib, a TKI targeting the angiogenesis, is currently considered the standard therapy in newly diagnosed, mRCC patients, who have a good-intermediate prognosis (Albiges et al., 2015). Nevertheless, pazopanib (PAZ), a multi-targeted TKI, has been recently approved in first-line mRCC treatment and in second-line, after treatment with cytokines. The approval comes from the results of the phase III, pivotal trial that compared PAZ to placebo in a heterogeneous population, including both therapy-naïve and cytokine-pretreated patients. PFS was significantly prolonged in the PAZ arm, in the first-line subset (median 11.1 vs. 2.8 months; *p* < 0.0001) as well as in the second-line setting (median, 7.4 vs. 4.2 months; *p* < 0.001) and in the overall cohort (median, 9.2 vs. 4.2 months; *p* < 0.0001); the median OS was 22.9 months with PAZ and 20.5 months with placebo, with an ORR of 30 vs. 3% with placebo (Sternberg et al., 2010).

A large non-inferiority study, the COMPARZ, compared PAZ to sunitinib in first-line, showing no statistically significant difference in terms of PFS, OS, and response rate (RR), with a slightly different toxicity profile in favor of PAZ (Motzer et al., 2013a). Subsequently, the PISCES (Patient Preference Study of PAZ vs. Sunitinib in Advanced or Metastatic Kidney Cancer) trial confirmed that PAZ had a better safety profile and was associated to a better quality of life, compared to sunitinib (Escudier et al., 2014).

Although some authors have questioned the results of the above-mentioned studies, the evidences are robust enough to support the use of PAZ in first-line as valuable alternative to sunitinib. To date there are not validated prognostic and predictive biomarkers of response, and none of the available studies has provided any straight, unbiased factor, which might guide the clinician in choosing PAZ rather than sunitinib, or vice versa. In the front-line setting, hence the decision is made exclusively on the basis of the safety profile and patients medical history; in particular, PAZ is generally preferred in patients with severe cardiovascular co-morbidities or poor performance status (D'Alterio et al., 2010, 2012; Sonpavde et al., 2012; Choueiri et al., 2013; Ravaud et al., 2013; D'Aniello et al., 2014; Cavaliere et al., 2015).

Several “real world” studies have showed the efficacy and safety of PAZ in unselected populations (Galvis et al., 2013; Matrana et al., 2013a; Jonasch et al., 2014; Rizzo et al., 2015; Vogelzang et al., 2015), we thought to further reinforce such evidences publishing our own experience with the drug.

## Materials and methods

### Patients

This is a mono-institutional, observational, retrospective study, which was carried out in the Department of Uro-Gynecological Oncology of the National Cancer Institute of Naples, Italy, after approval by the National Cancer Institute of Naples Institutional Board. The study was carried out in accordance with the recommendations of the local review board with written informed consent from all subjects. All subjects gave written informed consent in accordance with the Declaration of Helsinki. We retrieved from our archive the medical records of all the patients with mRCC, treated with PAZ, in first-line, from June 1st, 2012, when PAZ became available at our center, to March 1st 2016. To be eligible, patients were required to meet the following inclusion criteria: histologically confirmed mRCC, treatment with PAZ started between June 1st 2012 and March 1st 2016, aged ≥18 years. PAZ was given according to the conventional schedule of 800 mg daily. Last date of follow up was June 1st 2016.

### Statistical analysis

Descriptive statistics were used to describe baseline characteristics, treatment patterns and adverse events (AEs). Categorical variables are described by patient counts and percentages. PFS by treatment line of therapy was analyzed using Kaplan-Meier analyses and log rank comparisons Kaplan-Meier survival curves. The Cox proportional hazards model was used to test the effect of those variables that reached the statistically significance at univariate analysis, on survival outcomes in multivariate analyses. Hazard ratios (HRs) and 95% CIs were estimated, adjusting for age and gender.

The primary endpoints were progression free survival (PFS) and OS; the secondary endpoint included the objective response rate (ORR), the disease control rate (DCR), and the safety profile. The potential relationships between patients' characteristics, AEs and response were also explored. PFS was defined as the interval between the date of the first dose of PAZ and the date of the disease progression or death from any cause; disease progression was defined as radiological tumor progression according to Response Evaluation Criteria In Solid Tumors, RECIST (Vogelzang et al., 2015) version 1.1, or clinical progression, including death. AEs were graded according to Common Terminology Criteria for AEs version 4.0.

## Results

### Patient characteristics

From June 1st 2012 to March 1st 2016, 128 patients with newly diagnosed mRCC or recurrent RCC were seen in our department, 124 received a TKI, 45 (36.6%) were treated with PAZ, 38 (30.6%) were eligible for the final analysis.

In regards to demographics and baseline features, median age was 61 years old, most patients were male (57.9%), the 87.6% had a Karnofsky Performance Status (Karnofsky PS) ≤ 80%, more than half (73.6%), had two or more co-morbidities. In terms of disease characteristics, the 60.5% underwent to prior nephrectomy; few patients had a histology different from clear cell (15.8%); almost all (86.8%) had both bone and visceral metastases. According to Memorial Sloan-Kettering Cancer Center Score (MSKCC), the most of patients were classified as “intermediate” (63.1%) and “good risk” (13.1%), only 4 patients were “poor risk,” unfit for starting sunitinib or temsirolimus for multiple and specific co-morbidities (Table 1).

**Table 1:** **Baseline demographic and clinical patients' characteristics**.

		**Patient *n*** = **38**
Median age, years (range)	< 70, *n* (%)	61 (42–79)
	≥70, *n* (%)	17 (44.7%)
		21 (55.3%)
Sex	male *n* (%)	22 (57.9%)
	female *n* (%)	16 (42.1%)
PS Karnofsky, *n* (%)	100–90%	5 (13.1%)
	80–70%	27 (71.8%)
	≥60%	6 (15.8%)
Comorbidities, *n* (%)	0	4 (10.5%)
	1	6 (15.7%)
	2	17 (44.7%)
	≥3	11 (28.9%)
Hypertension (%)	yes	21 (55.3%)
	no	17 (44.7%)
Metastatic disease at diagnosis (%)	Yes	19 (50%)
	No	19 (50%)
MSKCC/Motzer Score^*^, *n* (%)	Favorable	5 (13.1%)
	Intermediate	24 (63.1%)
	Poor	4 (12.5%)
	Unknown	0
Prior nephrectomy, *n* (%)		23 (60.5%)
Time form diagnosis to treatment, months (range)		13 (1–96)
Hystology, *n* (%)	Clear cell carcinoma	32 (84.2%)
	Type I papillary	1 (2.6%)
	Type II papillary	2 (5.3%)
	Cromophobe	1 (2.6%)
	Sarcomatoid variant	2 (5.3%)
Number of metastatic sites, *n* (%)	< 6	23 (60.5%)
	≥6	15 (39.4%)
Most common metastatic sites, *n* (%)	Lung	21 (55.3%)
	Bone	18 (47.4%)
	Lymph nodes	16 (42.1%)
	Liver	4 (10.5%)
	Other	10 (26.3%)

*n, number of patients*.

* *Memorial Sloan-Kettering Cancer Center*.

### Clinical outcomes

**Figure 1:**
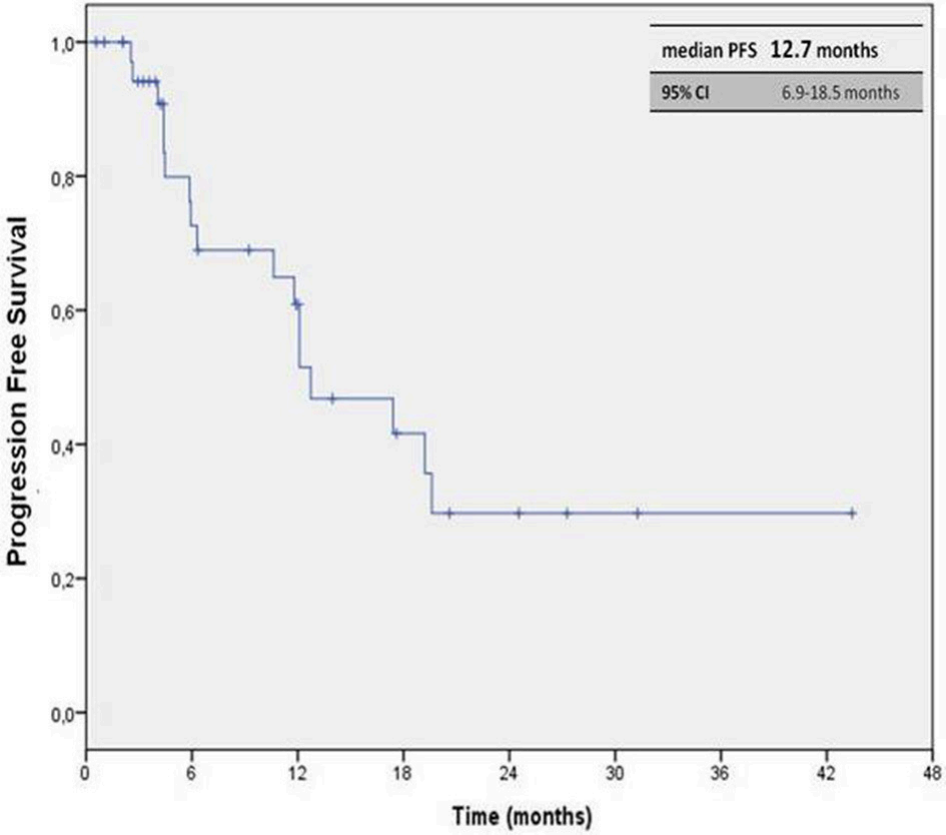
**Kaplan-Meier curve of median PFS in our cohort of patients treated with PAZ as first line therapy**.

**Figure 2:**
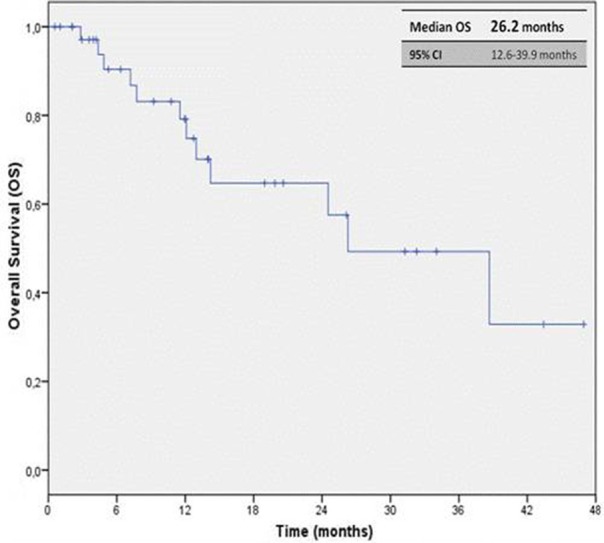
**Kaplan-Meier curve of median OS in our cohort of patients treated with PAZ as first line therapy**.

The median PFS (mPFS) was 12.7 months (95% CI, 6.9–18.5 months), with median OS (mOS) of 26.2 months (95% CI, 12.6–39.9 months; Figures 1, 2). The ORR, according to RECIST criteria version 1.1 (Eisenhauer et al., 2009), was 30.3%, with the 27.3% of patients achieving a partial response, and one patient having a complete response. The DCR was 72.7% (Table 2). The median time to best response was 12 weeks. Overall PAZ was well-tolerated, with no grade 4 toxicity. The most common AEs were hypertension occurring in the 40.6% of the cases, followed by dysthyroidism (28.9%) and diarrhea (15.8%). The incidence of the liver function tests (LFTs) abnormalities (elevations in levels of alanine transaminase [ALT], and/or aspartate transaminase [AST]) was 26.3%, all events were reversible with an average recovery of 10 days (Table 3). Some patients required a dose reduction due to toxicity (23.7%), but approximately the half of them (45%) was able to go back on the full dose after regression of the symptoms; none required drug discontinuation due to side effects (Table 4). Of note, six patients were given PAZ at 400/600 mg daily rather than 800 mg due to significant co-morbidities apparently without any effect on the outcome, reaching 20 months as maximum duration of treatment.

**Table 2:** **Objective response with PAZ in our study population**.

	**Patient n = 38**
**BEST RESPONSE, *n* (%)**	
CR	1 (3%)
PR	9 (27.3%)
SD	14 (42.4%)
PD	9 (27.3%)
Not available^*^	5 (15.5%)
ORR (CR + PR), *n* (%)	10 (30.3%)
DCR (CR + PR + SD) *n* (%)	24 (72.7%)

*n, number of patients; CR, complete response; PR, partial response; SD, stable disease; PD, progressive disease; ORR, objective response rate; DCR, disease control rate*.

* *not available at the time of the analysis*.

**Table 3:** **Adverse events of first-line PAZ in our study population**.

	**All grades**	**Grade 3/4**
Hypertension	13 (40.6%)	4 (10.5%)
Dysthyroidism	11 (28.9 %)	1 (2.6%)
Alteration of liver function	10 (26.3%)	6 (15.7%)
Diarrhea	6 (15.8%)	0
Mucositis	4 (13.1%)	1 (2.6%)
Fatigue	4 (10.5%)	0
Anemia	4 (10.5%)	1 (2.6%)

**Table 4:** **Dose modification and/or interruption due to adverse events**.

Dose reductions *n*, %	9 (23.7%)^*^
Treatment interruptions *n*, %	0

*n, number of patients*.

* *~45% of patients received a subsequent re-escalation of dose within 3 weeks*.

PFS appeared to not be influenced by number of co-morbidities (< 3 vs. ≥3), gender, Fuhrman grade and age (Table 5). A longer mPFS, that not reached the statistically significance, was seen in patients with only bone vs. visceral metastases vs. both (17.4 vs. 12.7 vs. 12.1 months; 95% CI, 1.6–26.4 months; *p* = 0.7). A worse mPFS was achieved in patients with liver metastases (mPFS 5.9 vs. 12.7 months; 95% CI, 3.5–18.4; *p* = 0.2) and with higher tumor burden (number of metastatic sites < 6 vs. ≥6) with mPFS 19.2 vs. 6.3 months (*p* = 0.08). Patients that gained a higher ORR and DCR had better outcomes in terms of both mPFS (HR = 0.05 [95% CI, 0.05–0.55], *p* = 0.01; HR = 0.10, [95% CI, 0.02–0.43, *p* = 0.002, respectively; Table 6A), but not in terms of mOS (*p* = 0.2; *p* = 0.35, respectively; Table 5). No statistically significant differences in mPFS was observed according to histology (*p* = 0.3; Table 5). As expected, the prior nephrectomy positively influenced the OS compared to no prior surgery (HR = 0.32 [95% CI, 0.09–1.07], *p* = 0.06; Table 6B). Worst OS was observed in patients aged ≥70 years old (HR = 6.91 [95% CI, 1.49–31.91], *p* = 0.01; Table 6B), but Karnofsky PS did not influence PAZ efficacy (HR = 1.65 [95% CI, 0.39–7.0], *p* = 0.5; Table 6B).

**Table 5:** **Univariate analysis of PFS and OS in our study population (***N*** = 38)**.

	**PFS**	**OS**
	***p*-value**	***p*-value**
**GENDER**	0.3	0.2
Male/Female		
**N**° **OF COMORBIDITIES**	0.8	0.8
< 3/≥3		
**HYSTOLOGY**	0.3	0.8
Clear Cells/Other		
**AGE**	0.4	0.003
< 70 years/≥70 years		
**KARNOFSKY PS**	0.2	0.02
< 80%/≥80%		
**PRIOR NEPHRECTOMY**	0.6	0.03
Yes/No		
**METASTATIC OF SITES**	0.08	0.4
≤ 5/≥6		
**DYSTHYROIDISM**	0.04	0.8
No/Yes		
**HYPERTENSION**	0.6	0.02
No/G1-2/G≥3		
**HEPATOTOXICITY**	0.6	0.8
G1-2/G≥3/No Toxicity		
**TUMOR RESPONSE RATE**	0.002	0.2
**DCR**	**0.0001**	**DCR**
DCR (CR + PR + SD)/PD		

*PFS, progression-free survival; OS, overall survival; CR, complete response; PR, partial response; SD, stable disease; PD, progressive disease; DCR, disease control rate; PS, performance status*.

**Table 6A:** **Multivariate adjusted Cox model for progression-free survival**.

	**Progression-free survival**		
	HR^*^	**(95% CI)**	***p*-value**
**DYSTHYROIDISM**			
Yes	0.12	(0.02–0.78)	0.02
**ORR**			
(RC + RP)	0.05	(0.05–0.55)	0.01
**DCR**			
(RC + RP + SD)	0.10	(0.02–0.43)	0.002

HR, hazard ratio; 95% CI: 95% confidence interval;

* *Multivariate Cox model adjusted for age and gender*.

**Table 6B:** **Multivariate adjusted Cox model for overall survival**.

	**Overall survival**		
	HR^*^	**(95% CI)**	***p*-value**
**AGE**			
≥70 years	6.91	(1.49–31.91)	0.01
**KARNOFSKY PERFORMANCE STATUS**			
< 80%	1.65	(0.39–7.0)	0.5
**NEPHRECTOMY**			
Yes	0.32	(0.09–1.07)	0.06
**HYPERTENSION**			
≥G3	0.22	(0.05–0.8)	0.03

HR, hazard ratio; 95% CI: 95% confidence interval;

* *Multivariate Cox model adjusted for age and gender*.

The impact of the hypertension seemed to be predictive of a prolonged survival, that was greater in patients that experienced a grade 3 event according to CTCAE v4.0 (HR = 0.22 [95% CI, 0.05–0.8], *p* = 0.03; Table 6B), but not in terms of mPFS (*p* = 0.6; Table 5). In addition, tumor response was influenced by hypertension tough four, among the five patients who developed grade 3 hypertension (80%), had PR. The hepatotoxicity not influenced the mPFS (*p* = 0.6) and OS significantly (*p* = 0.8; Table 5). The occurrence of a thyroid dysfunction (hypo-hyperthyroidism) during treatment with PAZ resulted in a prolonged mPFS (HR = 0.12 [95% CI, 0.02–0.78], *p* = 0.02; Table 6A) but not mOS (*p* = 0.8; Table 5).

### Outcome in non-clear cell histology

A small percentage (15.8%) of our population, 6 patients, had a histology different from clear cell. One patient had a type I papillary tumor; two had a type II papillary tumor; one had a chromophobe; two a sarcomatoid variant of renal cancer. PAZ has been active and well-tolerated with 3 patients achieving PR and 3 SD, the median duration of treatment in such subgroup has been 9.3 months, the mPFS of 10.6 months (95% CI, 4.4–16.8 months).

## Discussion

Randomized clinical trials are the gold standard to evaluate the efficacy of a new treatment within a specified patient population and the available international guidelines for physicians for treatment of mRCC essentially reflect the evidences for the different agents emerged from the respective clinical trial (AIOM, 2015; ESMO, 2015; NCCN, 2015). Nevertheless, the populations included in pivotal clinical trials are highly selected (no relevant co-morbidities, no poor performance status, no atypical metastases [e.g., brain metastases] or no histo-types different from clear cell) and generally have better outcomes compared to patients who are ineligible for such studies (Molina and Motzer, 2011; Heng et al., 2014). Therefore, it is not always simple to transfer the clinical trials results into clinical practice due to the large proportion of patients who are routinely treated in our clinics but would be judged ineligible in the pivotal trials. The optimization of the disease management should be based on clinical trials but also on real experiences (Schmidinger et al., 2013). Such registries, also known as “real world” or “real life” studies, providing insights into treatment patterns and outcomes, are emerging as a crucial tool for the clinicians because of a major reproducibility compared with randomized trials (Shek et al., 2012; Ta et al., 2013; Vaishampayan et al., 2014).

In terms of mPFS, we observed a median of 12.7 months, which is longer than the one reported in the COMPARZ (Motzer et al., 2013a) trial (8.4 months) and similar to the one detected in the pivotal phase III trial for the group of treatment-naïve patients (Sternberg et al., 2010). Looking at the different “real world” experiences, the mPFS was 8.5 months in the Vogelzang et al. (2015) analysis, 13.7 months in the MD Anderson Cancer Center study 17 (Matrana et al., 2013a), and 13.0 months in the Christie study (Galvis et al., 2013), in which the 74% of the patients receiving PAZ was treatment-naïve. The variations in the mPFS observed in these studies could have resulted from varying operational definitions of PFS such as different timing and frequency of disease assessment.

Comparing the data about survival, we noted that our median OS, 26.2 months, was longer than the ones observed in other studies, the pivotal trial (22.9 months), in the Christie study (19 months; Galvis et al., 2013), Vogelzang NJ et al analysis (22 months) (Vogelzang et al., 2015), and similar to the ones detected in the COMPARZ trial (28.4 months; Motzer et al., 2013a) and in the MD Anderson “real life” study (29.1 months; Matrana et al., 2013a). ORR and DCR appeared similar when compared to the outcomes of the randomized, phase III trial. We have been comforted by PAZ activity, therefore is not obvious to consider that patients with greater DCR and objective response obtained an improvement in OS and PFS. It is interesting to note that Karnofsky PS not influenced the OS, in contrast previous nephrectomy and an age ≤ 70 year old impact positively.

Overall our findings are very encouraging especially considering that the population was totally unselected, with a large proportion of frail patients, presenting more unfavorable prognostic factors (multiple co-morbidities, high tumor burden, worse PS and lower number of nephrectomies) compared to the available literature; our efficacy data are substantially consistent with those reported in both the prospective phase III trials and the retrospective analyses (Galvis et al., 2013; Matrana et al., 2013a,b; Motzer et al., 2013a,b; Sternberg et al., 2013; Jonasch et al., 2014; Vogelzang et al., 2015).

In regards to tolerability, we reported a safety profile slightly better compared with the other experiences. In our cohort, no grade 4 toxicity occurred and no permanent drug discontinuations due to toxicity were required. Furthermore, the rate of dose reductions due to AEs was 28%, but up to 45% received a subsequent re-escalation of dose.

The most frequent AE was hypertension observed in the 40.6% of the cases, such rate is similar to the ones observed in the COMPARZ (46%; Motzer et al., 2013a) and in the phase III pivotal trial (40%) (Sternberg et al., 2010), but superior to the one reported in the MD Anderson study (Matrana et al., 2013a) (21%) and in the analysis by Vogelzang et al. (2015) (27%). It is important to underline that about 25% of patients had poorly controlled hypertension at the times beginning PAZ, compared to the phase III trials. This medical condition was not related to a higher risk of cardiac toxicity during PAZ treatment, supporting its use even in this subset. The differences in hypertension incidence across the studies might have arisen from closer monitoring of vital signs during our study protocol according to the clinical trials. In contrast, the rates of fatigue (10.5%) and diarrhea (15.8%) were lower than previously reported: the MD Anderson study (Matrana et al., 2013a), 58 and 39%, respectively; Vogelzang analysis (Vogelzang et al., 2015), 56 and 52%, respectively; COMPARZ (Motzer et al., 2013a): 55 and 63%, respectively; Phase III trial: 19 and 52%, respectively (Sternberg et al., 2010). These differences in the incidence of fatigue are likely to be due both to the variability of the evaluation criteria adopted and to the retrospective nature of our data which probably led to an underestimation of the toxicities, especially the ones that are subjectively evaluated such as fatigue/asthenia.

The incidence of hepatic impairment was as expected (26.3%), the elevation of the liver function tests of grade 3 involved only 6 patients, and this event has been reversible and was not related to liver metastasis or other liver illness.

Six patients were treated with a lower dose of PAZ for concomitant diseases for the entire duration of treatment; we found that dose reduction was not associated with reduced efficacy, suggesting that the personalization of the drug schedule and the early management of the emerging toxicities represent crucial steps to maximize the therapeutic benefit.

We failed to identify predictive factors of response, although the occurrence of a thyroid dysfunction and grade 3 hypertension seemed to be related to a prolonged PFS and OS, respectively.

The use of PAZ out of clinical trial allowed us to evaluate its activity also in a subset of patients with histology other than clear cell, usually excluded from academic studies for their poor prognosis and limited sensibility to anticancer drugs. Our patients were not candidate for sunitinib and or temsirolimus treatment due to several specific co-morbidities. PAZ demonstrated to be still active also in this subset of patents with a mPFS of 10.6 months (95% CI, 4.4–16.8 months), data that are longer compared to those from pivotal phase III trial of sunitinib, and that will probably contribute to enrich the very few anecdotal literature data.

## Conclusions

Our results are consistent with those reported in prospective phase III trials and the published retrospective “real world” experiences. This analysis confirm the safety and efficacy of PAZ in first-line setting, both in frail patients with multiple co-morbidities and Karnofsky PS < 80% and in younger, healthier patients with number of metastatic sites < 6. Thyroid dysfunction and hypertension may represent prognostic factors for longer PFS and OS, respectively. More extensive data and larger sample are needed in order to appropriately guide real-world practice.

## Author contributions

SC, AC, GF, CD conceived the study, performed the statistical analysis, and wrote the paper. SR, CDP conceived the study, collected the data and wrote the paper. CC, MD, DS, GC, PMa, PMu, SiP, MB, SaP conceived the study and wrote the paper. GI, RP provided assistance with patients, wrote the paper and participate in the design of the study.

### Conflict of interest statement

The authors declare that the research was conducted in the absence of any commercial or financial relationships that could be construed as a potential conflict of interest. The reviewer FFM declared a shared affiliation, though no other collaboration, with several of the authors SC, SR, CDP, MD, AC, GI, RP, DS, GC, PMa, PMu, SiP, SaP, and GF to the handling Editor, who ensured that the process nevertheless met the standards of a fair and objective review.

## Abbreviations

RCC: renal cell carcinoma
TT: targeted therapy
OS: overall survival
TKI: tyrosine kinase inhibitor
VEGF: vascular endothelial growth factor receptor
PAZ: pazopanib.
