# Corrigendum: Pazopanib in Metastatic Renal Cancer: A “Real-World” Experience at National Cancer Institute “Fondazione G. Pascale”

**DOI:** 10.3389/fphar.2016.00468

**Published:** 2016-12-05

**Authors:** Sabrina C. Cecere, Sabrina Rossetti, Carla Cavaliere, Chiara Della Pepa, Marilena Di Napoli, Anna Crispo, Gelsomina Iovane, Raffaele Piscitelli, Domenico Sorrentino, Gennaro Ciliberto, Piera Maiolino, Paolo Muto, Sisto Perdonà, Massimiliano Berretta, Sandro Pignata, Gaetano Facchini, Carmine D'Aniello

**Affiliations:** ^1^Division of Medical Oncology, Department of Uro-Gynaecological Oncology, Istituto Nazionale Tumori IRCCS “Fondazione G. Pascale,”Naples, Italy; ^2^Department of Onco-Ematology Medical Oncology, S.G. Moscati Hospital of TarantoTaranto, Italy; ^3^Unit of Epidemiology, Struttura Complessa di Statistica Medica, Biometria e Bioinformatica, Istituto Nazionale Tumori IRCCS “Fondazione G. Pascale,”Naples, Italy; ^4^Pharmacy Unit, Istituto Nazionale Tumori IRCCS “Fondazione G. Pascale,”Naples, Italy; ^5^Division of Urology, Department of Uro-Gynaecological Oncology, Istituto Nazionale Tumori IRCCS “Fondazione G. Pascale,”Naples, Italy; ^6^Scientific Direction, Istituto Nazionale Tumori IRCCS “Fondazione G. Pascale,”Naples, Italy; ^7^Division of Radiation Oncology, Istituto Nazionale Tumori IRCCS “Fondazione G. Pascale,”Naples, Italy; ^8^Division of Medical Oncology–Centro di Riferimento OncologicoAviano, Italy; ^9^Oncology Unit, A.O.R.N. dei COLLI “Ospedali Monaldi-Cotugno-CTO,”Naples, Italy

**Keywords:** pazopanib, metastatic renal cancer, real world, angiogenesis inhibitors, treatment

In the original article, we have neglected to thank our funder, Novartis Farma S.p.A. The Funding Statement appears below.

## Funding

Novartis Farma S.p.A. supported publication of this article.

The authors apologize for this oversight. This error does not change the scientific conclusions of the article in any way.

### Conflict of interest statement

The author declares that the research was conducted in the absence of any commercial or financial relationships that could be construed as a potential conflict of interest.

